# Identification of altered immune cell types and molecular mechanisms in Alzheimer’s disease progression by single-cell RNA sequencing

**DOI:** 10.3389/fnagi.2024.1477327

**Published:** 2024-11-14

**Authors:** Hua Lin, Li Su, Daniel Mao, Grace Yang, Qi Huang, Yating Lan, Jingyi Zeng, Wenyi Song, Guining Liang, Qingyan Wei, Donghua Zou, Rongjie Li, Chanhua Zou

**Affiliations:** ^1^Department of Neurology, The Second Affiliated Hospital of Guangxi Medical University, Nanning, China; ^2^Department of Neurology, The Affiliated Hospital of Youjiang Medical University for Nationalities, Baise, China; ^3^Department of Biology, Pennsylvania State University, University Park, PA, United States; ^4^State College Area High School, State College, PA, United States; ^5^Department of Geriatrics, The Fifth Affiliated Hospital of Guangxi Medical University, Nanning, China; ^6^Department of Comprehensive Internal Medicine, Guangxi Medical University Caner Hospital, Nanning, China

**Keywords:** Alzheimer’s disease, immune cells, single-cell RNA sequencing, UFC1, monocytes, tlymphocytes

## Abstract

**Introduction:**

Alzheimer’s disease (AD) is a progressive neurodegenerative disorder characterized by gradual loss of cognitive function. Understanding the molecular mechanisms is crucial for developing effective therapies.

**Methods:**

Data from single-cell RNA sequencing (scRNA-seq) in the GSE181279 dataset and gene chips in the GSE63060 and GSE63061 datasets were collected and analyzed to identify immune cell types and differentially expressed genes. Cell communication, pseudotime trajectory, enrichment analysis, co- expression network, and short time-series expression miner were analyzed to identify disease-specific molecular and cellular mechanisms.

**Results:**

We identified eight cell types (B cells, monocytes, natural killer cells, gamma-delta T cells, CD8+ T cells, Tem/Temra cytotoxic T cells, Tem/Trm cytotoxic T cells, and mucosal-associated invariant T cells) using scRNA-seq. AD samples were enriched in monocytes, CD8+ T cells, Tem/Temra cytotoxic T cells, and Tem/Trm cytotoxic T cells, whereas samples from healthy controls were enriched in natural killer and mucosal-associated invariant T cells. Five co-expression modules that were identified through weighted gene correlation network analysis were enriched in immune- inflammatory pathways. Candidate genes with higher area under the receiver operating characteristic curve values were screened, and the expression trend of Ubiquitin-Fold Modifier Conjugating Enzyme 1 (UFC1) gradually decreased from healthy controls to mild cognitive impairment and then to AD.

**Conclusion:**

Our study suggests that peripheral immune cells may be a potential therapeutic target for AD. Candidate genes, particularly UFC1, may serve as potential biomarkers for progression of AD.

## Graphical Abstract

The flowchart of this study. AD, Alzheimer’s disease; AUC, area under receiver operating characteristic curve; DEGs, differentially expressed genes; NC, normal control; scRNA-seq, single cell RNA-sequencing.
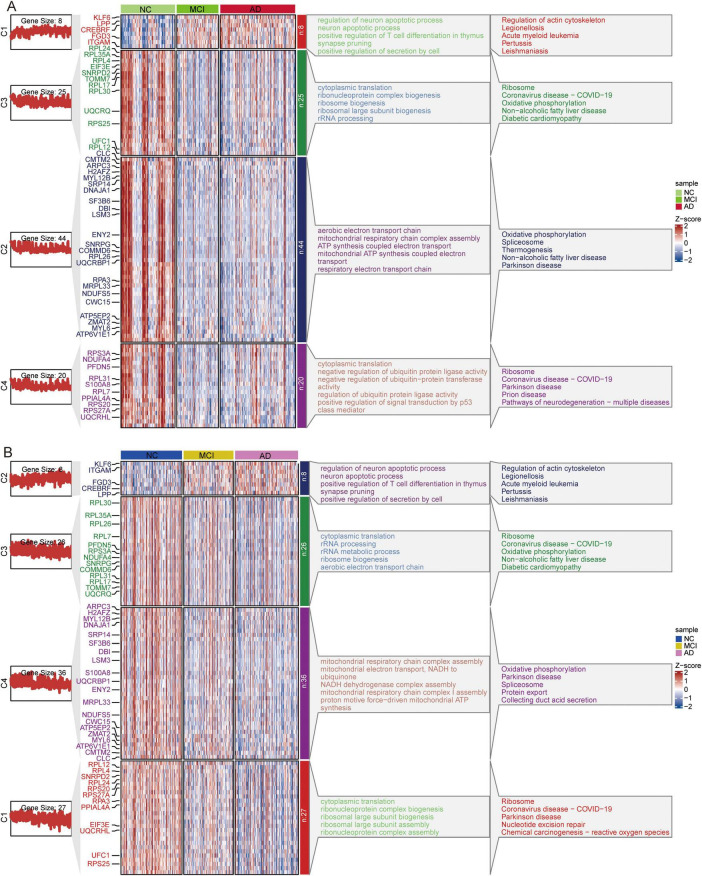

## Introduction

Alzheimer’s disease (AD) is a devastating neurodegenerative disorder that typically occurs in people over the age of 60 and is characterized by a gradual decline in cognitive functions, including memory, thinking, emotion, and behavior (Eissman et al., 2022; Zou D. et al., 2019; Zou et al., 2024). It is the most common cause of dementia, accounting for more than 65% of all dementia cases in elderly individuals (Ballard et al., 2011; Lin et al., 2022). While some treatment options are available for AD, its causes and mechanisms are not fully understood, underscoring AD as a significant focus in the scientific community (Zou et al., 2022; Jian et al., 2017).

The two main pathological hallmarks of AD are the accumulation of beta-amyloid proteins in amyloid plaques and the formation of neurofibrillary tangles, which are twisted fibers of tau protein that accumulate inside neurons (Gonzalez-Ortiz et al., 2023; Zou C. et al., 2019). These pathological changes lead to inflammation, oxidative stress, and damage to brain cells, eventually resulting in AD symptoms. Currently, there is no cure for AD, and the available treatments can only temporarily alleviate some of its symptoms (Zou et al., 2023).

Single-cell RNA sequencing (scRNA-seq) is a powerful tool for studying the molecular and cellular mechanisms underlying AD (Olah et al., 2020; Xu and Jia, 2021; Lu et al., 2024). AD is a complex and multifactorial disease that involves multiple cell types and molecular pathways (Xiong et al., 2021; Saura et al., 2023; Xie et al., 2024). scRNA-seq allows researchers to study individual cells, providing a more comprehensive understanding of the cellular and molecular changes associated with AD (Jian et al., 2021).

Targeting immune cells for AD as a potential therapeutic strategy has gained increasing attention in recent years (Hampel et al., 2020). The immune system plays a critical role in AD pathogenesis and the activation of immune cells, such as microglia and peripheral immune cells, contributes to disease progression (Luo et al., 2022; Ma et al., 2022). Studies have shown that peripheral immune cells are increased in AD patients and that immune cells in the periphery can influence the development and progression of AD (Bettcher et al., 2021).

Mild cognitive impairment (MCI) is often considered a transitional stage between normal aging and AD (Ritchie et al., 2014). Individuals with MCI are at increased risk of developing AD or other dementias (Davis et al., 2013). Studies have shown that the annual rate of conversion from MCI to AD is higher than that of cognitive decline in healthy older adults (Chandra et al., 2019). By analyzing and comparing the gene expression patterns from patients with MCI and AD, researchers can identify the molecular pathways that are switched towards AD and determine how these alterations contribute to AD progress.

To explore the molecular basis of peripheral immune cells and gene expression patterns, we used scRNA-seq data and transcriptomes from patients with AD and healthy controls to reveal the composition and proportions of immune cell types and identify key genes and pathways.

## Materials and methods

### Data collection

The GSE181279 (Xu and Jia, 2021) and GSE63063 (Sood et al., 2015) datasets were obtained from the Gene Expression Omnibus (GEO) database.^1^ GSE181279 includes scRNA-seq data of peripheral blood mononuclear cells (PBMCs) from three patients with AD and two cognitively normal controls (NC), based on the GPL24676 platform. GSE63063 is a superseries composed of GSE63060 and GSE63061 datasets. GSE63060 includes gene-chip datasets of blood samples from 49 patients with AD, 39 patients with mild cognitive impairment (MCI), and 67 NC based on the GPL6947 platform. GSE63061 included gene-chip datasets of blood samples from 40 patients with AD, 30 patients with MCI, and 72 NC, based on the GPL10558 platform.

### scRNA-seq cell clustering and differential analysis

During preprocessing, we used the Seurat R package (v3.1.2) to filter out empty droplets (those containing only ambient RNA) based on the criteria of expressing fewer than 200 genes. We also applied stringent thresholds for unique molecular identifier (UMI) count and mitochondrial gene content to exclude low-quality cells. Cells expressing more than 30% mitochondrial genes were filtered out to avoid cells undergoing apoptosis or stress. Additionally, we applied a lower UMI threshold of 200 and an upper threshold of 8,000 genes. After normalizing the data using the Seurat R package (v3.1.2), the FindClusters function was used to identify the major clusters. Subsequently, t-distributed stochastic neighbor embedding (tSNE) (Pont et al., 2019) was used to visualize major clusters. Cell types were defined based on the reported marker genes (Supplementary Table 1) and differential expression analysis of unique markers. Intercellular communication was analyzed using the CellChat R package (Jin et al., 2021). Pseudotime trajectory analyses were performed using monocle2 R package (Qiu et al., 2017). The contribution of cells to AD was calculated using the Seurat R package.

Differences between AD and NC in cell types were analyzed using the Seurat R package. Differentially expressed genes (DEGs) were identified at *P* < 0.05. Differences between cell types were analyzed using the limma R package (Ritchie et al., 2015). Enrichment analysis of biological processes (BP) in the Gene Ontology and Kyoto Encyclopedia of Genes and Genomes (KEGG) for DEGs was performed using the ClusterProfiler R package (Yu et al., 2012).

### Construction of co-expression network

The co-expression networks of the 2000 most variable genes were constructed using scRNA-seq with weighted gene correlation network analysis (WGCNA) (Langfelder and Horvath, 2008) and high-dimensional WGCNA. We constructed meta-cells for each cell and normalized their expression matrix. The soft threshold power was calculated, and the optimal β was selected to obtain a scale-free network. Then the adjacency matrix was transformed into a topological overlap matrix (TOM) and the co-expression network was constructed. Module eigengenes (MEs) were calculated to represent module expression levels. The correlation between genes and module eigengenes was calculated to obtain eigengene-based connectivity (kME) and to identify highly connected genes (hub genes) in each module. The top 25 hub genes in each module were scored using the moduleexprscor function. Correlations between the modules and cell types were calculated using Pearson’s correlation.

### Gene-chip data processing

DEGs between the AD and NC groups were analyzed using the limma R package with a permutation-based *P*-value of < 0.05. Common DEGs were obtained by intersectional analysis of DEGs that were up - or downregulated in both the GSE63060 and GSE63061 datasets.

Enrichment analyses of the Gene Ontology and KEGG databases for common DEGs were performed using Metascape.^2^ Gene set enrichment analysis (GSEA) was performed using the clusterprofiler R package to detect which gene sets were significantly enriched in AD. Adjusted *P* < 0.05 were considered statistically significant. The area under the receiver operating characteristic curve (AUC) values were calculated using the pROC package (Robin et al., 2011) for both the GSE63060 and GSE63061 datasets. Common DEGs with the top ten AUC values were selected as candidate genes.

### Short time-series expression miner (STEM)

We performed differential analyses between AD and MCI or between MCI and NC samples. The generated genes were provided as inputs to STEM (Ernst and Bar-Joseph, 2006) to observe variations in gene expression. Hierarchical clustering was used to observe activated or inhibited variation trends in the KEGG pathways.

## Results

### Single-cell gene expression profiles reveal major immune cell types in AD

Graphical Abstract highlights the experimental design, including the datasets used (GSE181279, GSE63060, and GSE63061) and their respective contributions to identifying immune cell types and gene expression profiles in AD and normal controls. The cell clustering based on scRNA-seq data is depicted, illustrating the differences in immune cell populations between AD patients and controls. The identification of key DEGs and the co-expression network analysis are also represented, emphasizing the role of immune cells in AD progression.

**FIGURE 1:**
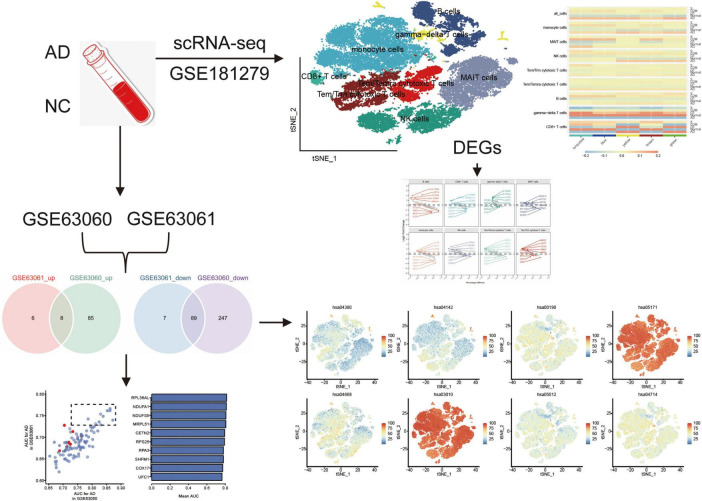
Characterization of cell populations in AD with scRNA-seq profiling. **(A)** The T-distributed stochastic neighbor embedding (tSNE) plot showing major cell types. **(B)** The tSNE plot showing subtypes of immune cells. **(C)** Dot plot showing average expression of marker genes of immune cell types. The color represents the average expression level of marker genes. **(D)** The tSNE plot showing subtypes of T cells. **(E)** The tSNE plot showing all cells in the AD and normal control groups. **(F)** Proportion of cell types in AD and normal control samples. AD, Alzheimer’s disease.

**FIGURE 2:**
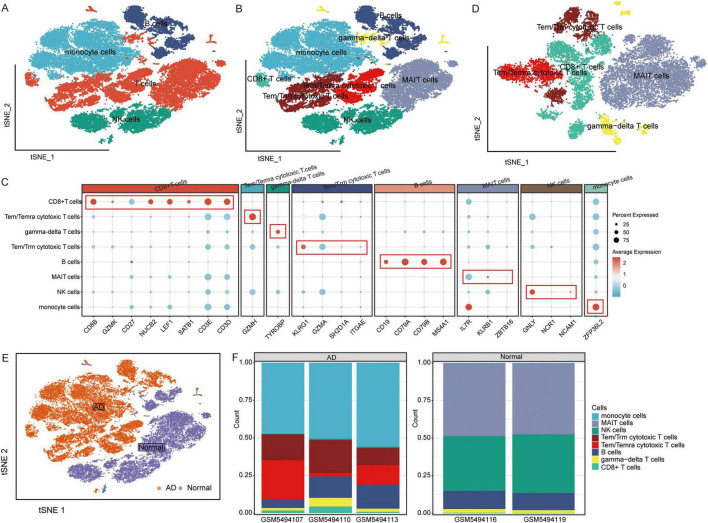
Single cell immune landscape in ARDS and healthy controls. **(A)** Cellular interaction number and strength. **(B)** Pseudotime trajectory analysis of major immune cells. **(C)** Heatmap of gene expression in immune cells of branches.

The raw GSE181279 dataset was read using the Seurat R package, and 22,776 individual cells in AD and 14,074 individual cells in normal tissue were obtained (Supplementary Figure 1). This was followed by quality control leading to 21,791 high-quality individual cells and 13,877 individual cells from normal individuals identified (Supplementary Figure 2). Using graph-based tSNE, we identified 13 clusters of major immune cells containing 35,668 total cells. Subsequently, we annotated 13 cell clusters with marker genes for major cell types and found that they were annotated as B cells, T cells, monocytes, and natural killer (NK) cells (Figures 1A, C). The distribution of T cells was clear and loose. Thus, we subdivided the T cell population and identified five T cell subtypes: gamma-delta T cells, CD8+ T cells, Tem/Temra cytotoxic T cells, Tem/Trm cytotoxic T cells, and mucosal-associated invariant T (MAIT) cells (Figures 1B–D). Different cell types were enriched in patients with AD and NC. Monocytes, CD8+ T cells, Tem/Temra cytotoxic T cells, and Tem/Trm cytotoxic T cells were enriched in patients with AD, whereas MAIT and NK cells were enriched in NCs (Figure 2E). Cell type proportions were significantly different between AD and NC, with monocytes, Tem/Temra cytotoxic T cells, and Tem/Trm cytotoxic T cells predominantly present in the AD samples, and MAIT cells, NK cells, and B cells predominantly present in the NC samples (Figure 1F).

### CellChat and cellular trajectory based on scRNA-seq

We sought to explore the communication networks between immune cells. The interactions between Tem/Temra cytotoxic T cells, Tem/Trm cytotoxic T cells, and CD8 cells were stronger, and CD8 cells were signal recipients (Figure 2A). Three cell branches were identified in the pseudotime analysis: gamma-delta T cells and CD8+ T cells concentrated at the end of branch 1, monocytes concentrated at the end of branch 2, and Tem/Temra cytotoxic T cells and Tem/Trm cytotoxic T cells bifurcating into branch 3 (Figure 2B). The gene expression of immune cells in these branches is shown in Figure 2C. These results revealed the potential transcriptional heterogeneity between cell types.

### Identification of co-expression network using WGCNA

**FIGURE 3:**
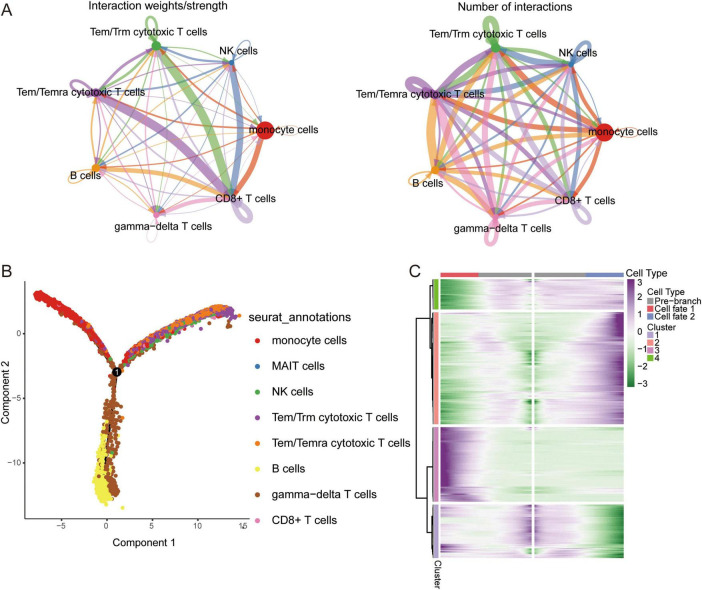
Co-expression modules and their association with immune cells. **(A)** Different soft-thresholding for screening scale-free network. **(B)** Hierarchical clustering tree of 5 modules of co-expression. **(C)** Score of each module in immune cells. The color represents the score level of top 25 hub genes. **(D)** Top 10 hub genes within individual modules were determined by kME values. ME, module eigengenes. **(E)** Correlation between modules and immune cells in different clinical traits. *P < 0.05, **P < 0.01, ***P < 0.001. **(F)** Differential condition of cell types in each module. *P < 0.05, **P < 0.01, ***P < 0.001.

To explore the co-expression networks of genes in immune cell types, we performed WGCNA. The power parameter range of 1–30 was filtered the power of β = 8 (scale-free R2 = 9) was used as the optimal screening soft threshold to construct a scale-free network and obtain five co-expression modules (Figures 3A, B). The modules spanned multiple cell populations by scoring the top 25 hub genes of each module, then mapping them to single cells (Figure 3C). We identified the top 10 hub genes in each module (Figure 3D). Among these modules, yellow (M3) and green (M5) modules showed significant positive correlations with CD8+ T cells and significant negative correlations with gamma-delta T cells in AD, while turquoise module (M1) showed significant positive correlations with monocytes and MAIT cells and significant negative correlations with CD8+ T cells, gamma-delta T cells, and B cells in AD (Figure 3E). Moreover, all immune cells were significantly different in the M1 group (Figure 3F).

### Differentially expressed genes and biological roles

**FIGURE 4:**
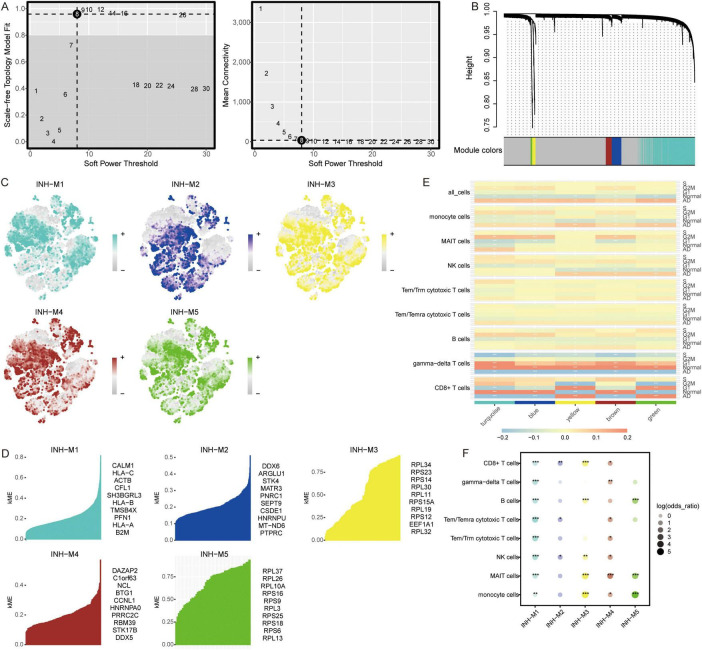
Contribution of immune cell types, analysis of differentially expressed genes, and metabolic pathway enrichment analysis in AD. **(A)** Contribution of different immune cell subtypes to AD. **(B)** Volcano plot of differentially expressed genes in various immune cell types between AD and control samples. **(C)** Metabolic pathway enrichment analysis based on differentially expressed genes. **(D)** Quantitative analysis of metabolic pathways in different immune cell types.

We investigated the contribution of immune cells in AD. The results indicated that MAIT cells had the highest contribution to AD, followed by B cells and NK cells (Figure 4A). By comparing gene expression changes between AD samples and normal controls, we found that MAIT cells and monocytes were primarily upregulated in AD (Figure 4B). Enrichment analysis of DEGs in various cell types revealed that the DEGs were mainly enriched in growth hormone synthesis, secretion, and action (Figure 4C). In the quantitative analysis, we found that Butanoate metabolism was significantly activated in MAIT cells (Figure 4D).

**FIGURE 5:**
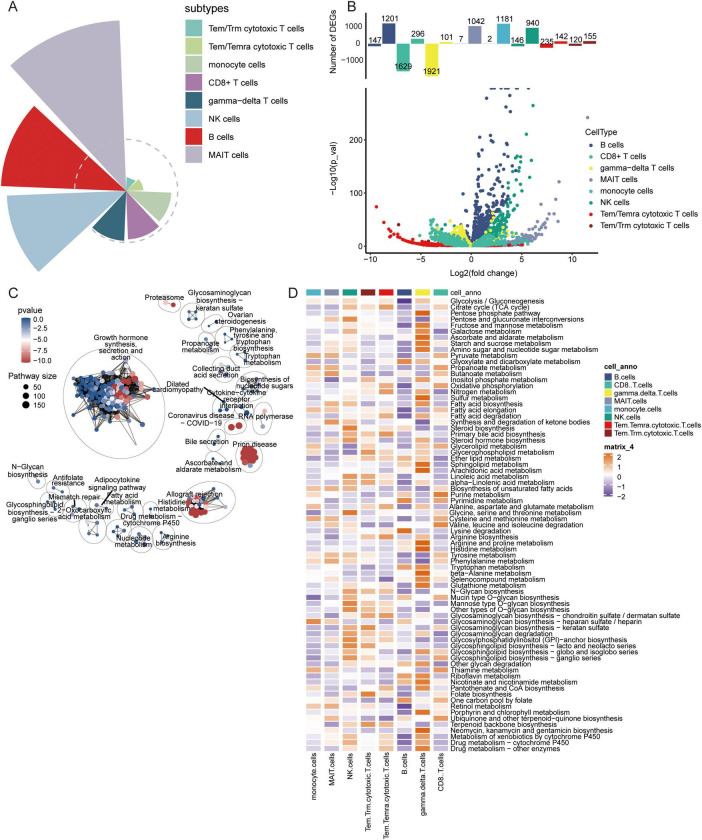
Identification of differentially expressed genes and biological roles of immune cells based on scRNA-seq. **(A)** Differentially expressed genes in each cell type compared to others. The top 5 up- or downregulated expressed genes are labeled. **(B)** Biological processes of differentially expressed genes in all cell types. The size represents the count of cells. The color represents the FDR. **(C)** KEGG pathways of differentially expressed genes in all cell types. The size represents the count of cells. The color represents the FDR. FDR, false discovery rate.

In addition, to explore the disease mechanism in patients with AD from a molecular perspective, we analyzed the genes in immune cells identified by scRNA-seq for differential expression. We identified 1683 DEGs in B cells, 951 DEGs in CD8+ T cells, 1407 DEGs in gamma-delta T cells, 250 DEGs in MAIT cells, 1615 DEGs in monocytes, 644 DEGs in NK cells, 1595 DEGs in Tem/Temra cytotoxic T cells, and 1582 DEGs in Tem/Trm cytotoxic T cells (Figure 5A). Enrichment analysis revealed that these DEGs were enriched for regulation of T cell proliferation, B cell activation, and inflammatory responses (Figure 5B). In KEGG pathway analysis, we found that the DEGs were mainly enriched for ribosomes, Parkinson’s disease, and AD (Figure 5C). Of these, MAIT cells are involved in the fewest signaling pathways. However, all are implicated in the nervous system.

**FIGURE 6:**
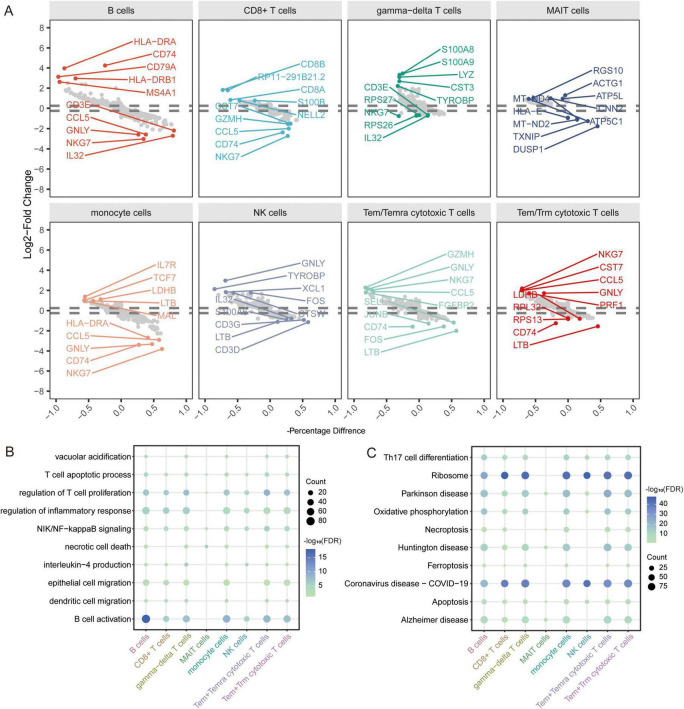
Identification of differentially expressed genes and biological roles of immune cells based on gene-chip data. **(A)** Differentially expressed genes between AD and normal controls in the GSE63060 dataset. The top 3 up- or downregulated expressed genes are labeled. **(B)** Differentially expressed genes between AD and normal controls in GSE63061 dataset. The top 3 up- or downregulated expressed genes are labeled. **(C)** Common DEGs were screened with the intersection of up-expressed genes or down expressed genes in both datasets. **(D)** Functional enrichment analysis of common DEGs through Metascape.

Notably, we identified 110 DEGs from the GSE63060 dataset (Figure 6A) and 429 DEGs from the GSE63061 dataset (Figure 6B). Analyzing the upregulated and downregulated DEGs separately, we found eight DEGs that were upregulated and 89 DEGs that were downregulated in both GSE63060 and GSE63061. Both were considered common DEGs (Figure 6C). Enrichment analysis showed that common DEGs were involved in SRP-dependent co-translational proteins targeting the membrane, oxidative phosphorylation, and ribonucleoprotein complex biogenesis (Figure 6D).

### Candidate genes and pathways in AD

**FIGURE 7:**
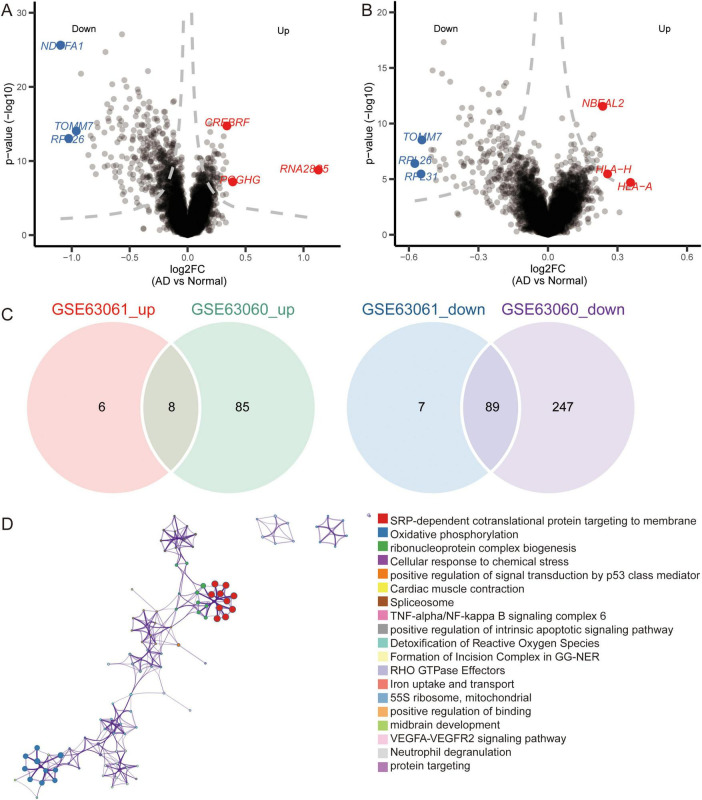
Identification of candidate genes. **(A)** AUC values of common DEGs in GSE63060 and GSE63061 datasets. Red represents high expression and blue represents low expression in AD. AUC, area under receiver operating characteristic curve. **(B)** Expression of candidate genes in 8 immune cells. The color represents the expression levels. **(C)** Violin plots showing the expression of candidate genes in 8 immune cells. **(D)** Expression of candidate genes in AD and normal controls in GSE63060 and GSE63061 datasets. ***P < 0.001. AD, Alzheimer’s disease.

We further extracted candidate genes (CETN2, COX17, MRPL51, NDUFA1, NDUFS5, RPA3, RPL36AL, RPS25, SHFM1, and UFC1) with high AUC values in both GSE63060 and GSE63061 (Figure 7A). RPS25 and RPL36AL were highly expressed in the eight immune cell types identified by scRNA-seq (Figures 7B, C). Interestingly, all candidate genes exhibited decreased expression in AD patients compared to normal controls in both the GSE63060 and GSE63061 datasets (Figure 7D).

**FIGURE 8:**
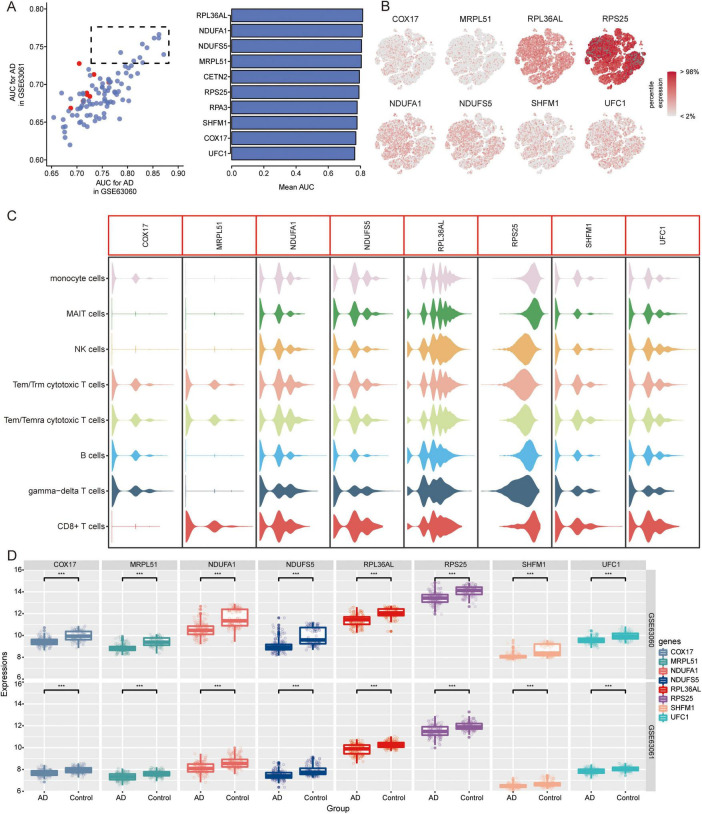
Analysis of pathways in AD and immune cells. Top 5 activated or inhibited intersecting KEGG signaling pathways in GSEA in GSE63060 **(A)** and GSE63061 **(B)** datasets. NES, normalized enrichment score. **(C)** Enrichment of pathways in 8 immune cells. The color represents the enrichment levels.

In addition, we analyzed the KEGG pathways in AD of GSE63060 (Figure 8A) and GSE63061 (Figure 8B) using GSEA. The results showed that in AD patients, osteoclast differentiation, lysosomes, TNF signaling pathway, JAK-STAT signaling pathway, growth hormone synthesis, secretion, and action were activated; while ribosomes, oxidative phosphorylation, coronavirus disease-COVID-19, Parkinson’s disease, and thermogenesis were inhibited. We found that ribosomes (hsa03010) and coronavirus disease-COVID-19 (hsa05171) were mainly enriched in the eight immune cell types identified by scRNA-seq (Figure 8C).

### Patterns of genes and signaling pathways in AD progression

**FIGURE 9:**
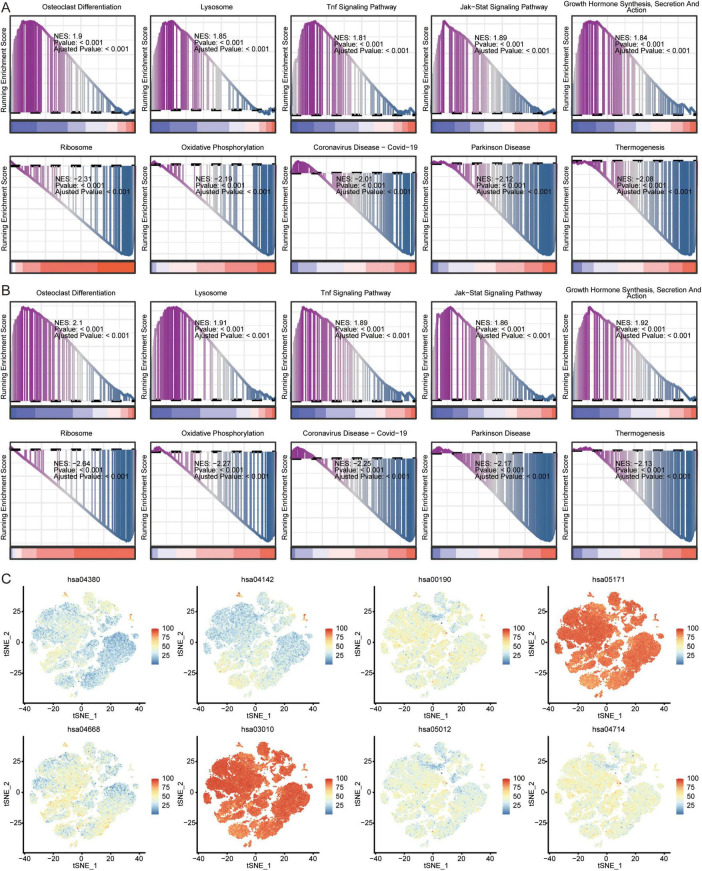
Expression trends of genes in STEM analysis. Genes with the same expression trend in GSE63060 **(A)** and GSE63061 **(B)** datasets. AD, Alzheimer’s disease; NC, normal control.

Temporal expression analysis was performed using STEM software of the differentially expressed genes between AD and MCI and between MCI and controls to further explore the expression patterns of genes in the progression of AD. We found that the expression trends of the 13 genes in the course of the normal control to MCI and AD were consistent in the GSE63060 (Figure 9A) and GSE63061 (Figure 9B) datasets. Among these 13 genes, UFC1 was identified as one of the candidate genes.

**FIGURE 10:**
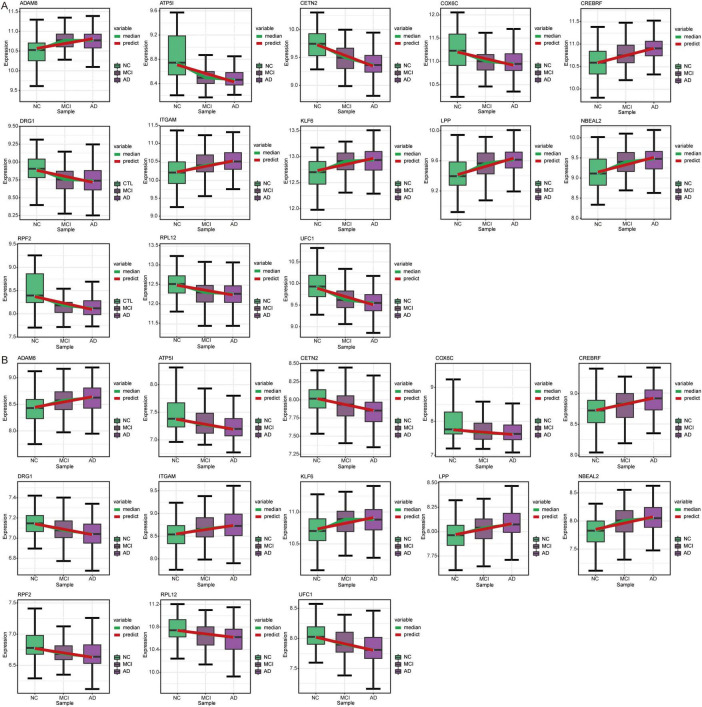
Clustered Heatmap of common DEGs and signaling pathways in AD, MCI, and NC groups. Expression heatmap of common DEGs and their major biological functions involved in GSE63060 **(A)** and GSE63061 **(B)** datasets. AD, Alzheimer’s disease; MCI, mild cognitive impairment; NC, normal control.

Cluster heatmap analysis of common DEGs in GSE63060 (Figure 10A) and GSE63061 (Figure 10B) revealed that the genes upregulated in AD progression were mainly involved in the regulation of neuronal apoptotic processes and the actin cytoskeleton. In contrast, the genes downregulated in AD progression were mainly involved in ribosome and oxidative phosphorylation.

## Discussion

scRNA-seq is a powerful tool for studying the molecular and cellular mechanisms of AD. Its applications are expected to accelerate the development of new therapies and diagnostic tools for this devastating disease (Murdock and Tsai, 2023). In this study, we identified eight immune cell types with significantly different proportions between the AD and NC groups. We also explored the co-expression networks of DEGs among immune cells. This study provides insights into the molecular mechanisms underlying AD and suggests potential therapeutic targets for the disease.

Using tSNE, we identified 27 clusters of immune cells, which were subsequently annotated with marker genes for major cell types. The composition of immune cell subsets is variably altered in patients with AD compared to NCs. Interestingly, we found that the AD and NC groups were enriched in different cell types. Previous studies have found increased numbers of CD8 + T cells in the postmortem brain tissue of patients with AD, which was also validated in a murine APP/PS1 amyloidosis model (Unger et al., 2020). TEM (effector memory T cells) and TEMRA (CD45RA+ effector memory T cells) carry the greatest amounts of perforin and Fas ligand, with their numbers increasing after viral infection (Shen et al., 2010). The number and cytotoxic activity of blood NK cells were decreased in patients with AD compared to those in control subjects, which may be related to tissue transfer and neurogenic innervation of NK cells (Qi et al., 2022). Consistent with the findings of this study, MAIT cell abundance was also reduced in the AD group compared to the healthy control group (Qian et al., 2022). MAIT cells have the greatest contribution to AD, indicating that they may have important immune regulatory functions in the pathological process of AD. In quantitative analysis of metabolic pathways, we found significant activation of Butanoate metabolism in MAIT cells. Changes in metabolism may affect cell function and survival, thereby affecting the progression of AD.

CellChat can be used to identify and visualize the cell-cell communication networks involved in AD. These findings suggest that the dysregulation of immune cells may play a role in the pathogenesis of AD. Communication between Tem/Temra cytotoxic T cells, Tem/Trm cytotoxic T cells, and CD8+ cells was strong. This may be related to the fact that these three cells share common marker genes (Xiong et al., 2023; Sallusto et al., 2004). These interactions suggest that cytotoxic T cells, known for their role in immune surveillance and clearance of damaged cells, may contribute to the neurodegeneration observed in AD. Increased cytotoxicity may drive inflammation and exacerbate neuronal damage, implicating these cells in the progression of the disease. Pseudotime analysis further supported the idea that immune cells follow distinct developmental pathways during AD progression. These findings provide evidence for the temporal and functional heterogeneity of immune cells in AD. Given immune cells increased activation and altered communication in AD, modulating the activity of specific immune cell populations - particularly cytotoxic T cells and monocytes−could help reduce neuroinflammation and slow the progression of the disease.

WGCNA allows the identification of groups of genes that are highly correlated in their expression patterns across different cell types. Correlation analysis suggests that genes in yellow (M3) and green (M5) modules are highly active in CD8+ T cells but less active in gamma-delta T cells in the context of AD. Genes in turquoise (M1) are highly active in monocytes and MAIT cells, but less active in CD8+ T cells, gamma delta T cells, and B cells in the context of AD. The enriched biological functions of DEGs in different immune cell types were regulation of the immune inflammatory response, ribosomes, and AD. This suggests a potential link between these immune cell types and AD development (Zhou et al., 2021). We noted that MAIT cells, in particular, are involved in pathways implicated in the nervous system. This finding is important because it suggests that MAIT cells may play a crucial role in neuroinflammation and neurodegeneration observed in AD (Wyatt-Johnson et al., 2023; Elkjaer et al., 2022; Liang et al., 2022).

On top of that, we identified candidate genes based on the diagnostic efficacy. We performed temporal expression analysis to investigate the progression of AD using STEM software and found that the expression trends of 13 genes were consistent in both the GSE63060 and GSE63061 datasets. Among these 13 genes, UFC1 (Ubiquitin-Fold Modifier Conjugating Enzyme 1) was identified as a candidate gene, exhibiting a trend of decreasing expression from NC to MCI and then to AD. UFC1 is downregulated in AD compared to controls (Madrid et al., 2021). UFC1 is involved in ubiquitination as an E2 conjugating enzyme and interacts with neuronal cell adhesion molecules in neurological diseases (Nahorski et al., 2018; Liu et al., 2015). However, its role in AD remains poorly understood.

This study has several limitations that should be considered. Firstly, this study was primarily focused on immune cells thus, excluding other cell types that may also be significantly involved in AD pathology. Furthermore, this study used data from publicly available datasets and did not include new experimental data. The sample size was relatively small for scRNA-seq, which may also limit the generalizability of the findings. Moreover, this study did not address the causes behind the observed differences in immune cell populations between AD and NC samples. We also acknowledged that transcriptional changes do not always correlate directly with protein expression, and therefore, additional validation steps are necessary. In future studies, we plan to use techniques such as enzyme-linked immunosorbent assay or Western blotting to assess UFC1 protein levels in peripheral blood samples from AD patients, MCI patients, and normal controls. Further studies are needed to investigate the mechanisms underlying these differences. Consequently, the findings should be interpreted with caution, and further studies are needed to confirm and expand these results.

## Conclusion

Overall, the present study identified eight immune cell types at the single-cell level and explored the cell communication and co-expression networks of genes in these immune cell types. Candidate genes, particularly UFC1, may serve as potential biomarkers for AD progression. This study reveals the potential transcriptional heterogeneity between immune cell types and provides insights into the molecular mechanisms underlying AD.

## Data Availability

The original contributions presented in the study are included in the article/Supplementary material, further inquiries can be directed to the corresponding authors.

## Footnotes

1
https://www.ncbi.nlm.nih.gov/gds

2
https://metascape.org/gp/#/main/step1
