# Supplementary material for: Identification of altered immune cell types and molecular mechanisms in Alzheimer’s disease progression by single-cell RNA sequencing
Source: Front Aging Neurosci. 2024 Nov 14;16:1477327. doi: 10.3389/fnagi.2024.1477327 (PMC11602448; doi:10.3389/fnagi.2024.1477327)
Supplement: Supplementary file 1 [file Data_Sheet_1.pdf]

Supplementary Material

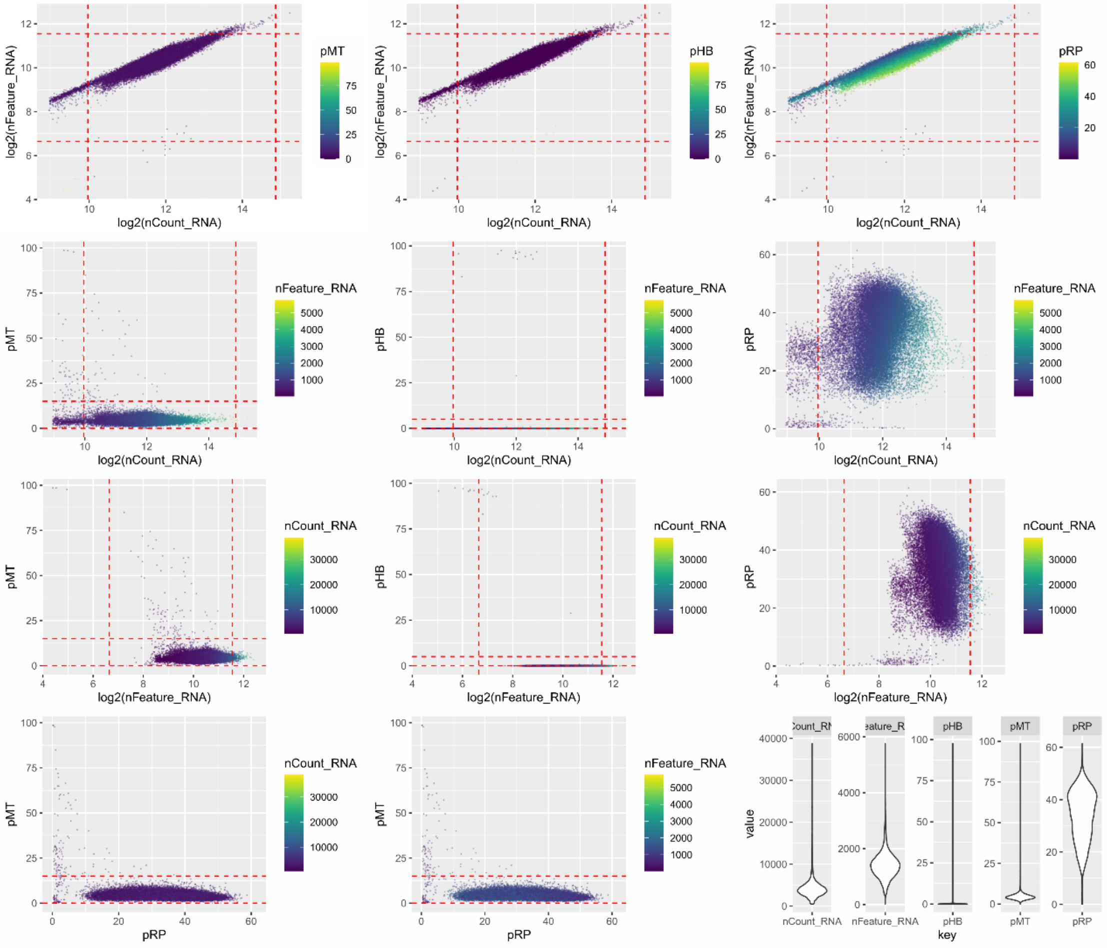

Figure S1. Total cells obtained from samples of AD and normal control before quality control. Including the range of detected gene numbers, sequencing counts, and percentage of mitochondrial sequencing counts per cell.

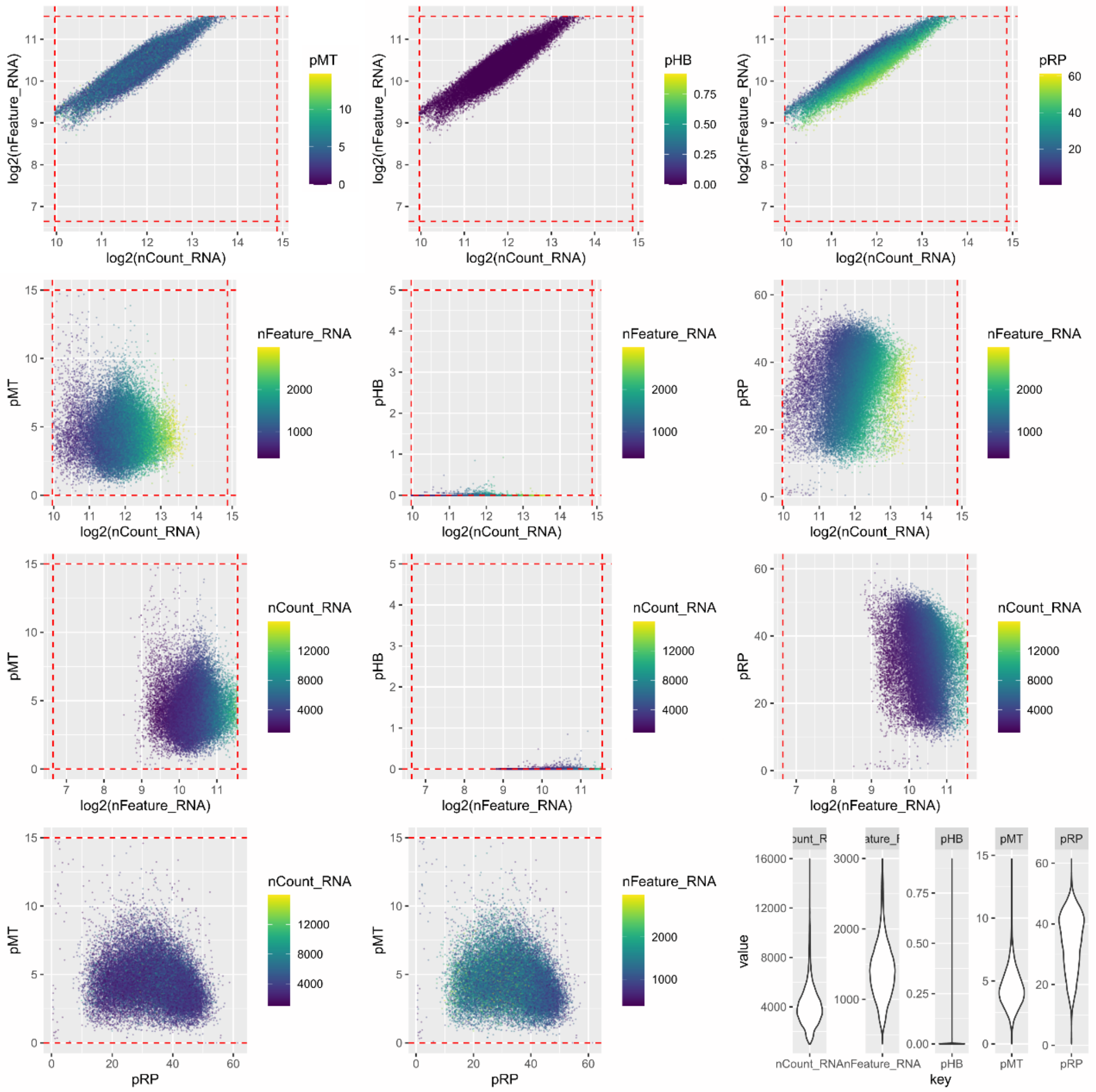

Figure S2. Total cells obtained from samples of AD and normal control after quality control. Including the range of detected gene numbers, sequencing counts, and percentage of mitochondrial sequencing counts per cell.

Table S1. Reported main marker genes to identify major cell types.

CD8+ T cells	CD8B, GZMK, CD27, NUCB2, LEF1, SATB1, CD3E, CD3D
gamma-delta T cells	TYROBP
MAIT cells	IL7R, KLRB1, ZBTB16
Tem/Temra cytotoxic T cells	GZMH
Tem/Trm cytotoxic T cells	KLRG1, ITGAE, GZMA, SH2D1A
B cells	CD19, CD79A, CD79B, MS4A1
NK cells	GNLY, NCR1, NCAM1
monocyte cells	ZFP36L2
